# Does polycystic ovarian morphology influence the response to treatment with pulsatile GnRH in functional hypothalamic amenorrhea?

**DOI:** 10.1186/s12958-016-0159-8

**Published:** 2016-04-29

**Authors:** Agathe Dumont, Didier Dewailly, Pauline Plouvier, Sophie Catteau-Jonard, Geoffroy Robin

**Affiliations:** Service de Gynécologie Endocrinienne et de Médecine de la Reproduction, Hôpital Jeanne de Flandre, Centre hospitalier régional universitaire de Lille, CHRU, Avenue Eugène Avinée, 59037 Lille, France

**Keywords:** Functional hypothalamic amenorrhea, Polycystic ovarian morphology, Pulsatile GnRH therapy, Excessive ovarian response, Pregnancy

## Abstract

**Background:**

Pulsatile GnRH therapy is the gold standard treatment for ovulation induction in women having functional hypothalamic amenorrhea (FHA). The use of pulsatile GnRH therapy in FHA patients with polycystic ovarian morphology (PCOM), called “FHA-PCOM”, has been little studied in the literature and results remain contradictory. The aim of this study was to compare the outcomes of pulsatile GnRH therapy for ovulation induction between FHA and “FHA-PCOM” patients in order to search for an eventual impact of PCOM.

**Methods:**

Retrospective study from August 2002 to June 2015, including 27 patients with FHA and 40 “FHA-PCOM” patients (85 and 104 initiated cycles, respectively) treated by pulsatile GnRH therapy for induction ovulation.

**Results:**

The two groups were similar except for markers of PCOM (follicle number per ovary, serum Anti-Müllerian Hormone level and ovarian area), which were significantly higher in patients with “FHA-PCOM”. There was no significant difference between the groups concerning the ovarian response: with equivalent doses of GnRH, both groups had similar ovulation (80.8 vs 77.7 %, NS) and excessive response rates (12.5 vs 10.6 %, NS). There was no significant difference in on-going pregnancy rates (26.9 vs 20 % per initiated cycle, NS), as well as in miscarriage, multiple pregnancy or biochemical pregnancy rates.

**Conclusion:**

Pulsatile GnRH seems to be a successful and safe method for ovulation induction in “FHA-PCOM” patients. If results were confirmed by prospective studies, GnRH therapy could therefore become a first-line treatment for this specific population, just as it is for women with FHA without PCOM.

## Background

Functional Hypothalamic Amenorrhea (FHA) is one of the most common causes of secondary amenorrhea [1–3]. FHA is due to a chronic energy deprivation, mostly caused by significant weight loss, severe food restriction and/or excessive exercise. This negative energy balance leads to a reduced frequency of the gonadotropin-releasing hormone (GnRH) pulses, which is responsible for gonadotropin insufficiency and results in anovulation and hypoestrogenism [2]. It has been demonstrated that pulsatile GnRH therapy is the best treatment to induce ovulation and pregnancy in women with FHA [4, 5], but it is available in only few countries worldwide. In women with FHA, by restoring a GnRH secretion and its pulsatility, this therapy results almost in 95 % pregnancy rate in 6 months [6, 7].

Polycystic ovarian morphology (PCOM) is the third criterion of the Rotterdam classification for Polycystic ovarian syndrome (PCOS) [8, 9]. Its definition essentially lies on an excessive number of antral follicles per ovary (FNPO) ≥ 12 and/or ovarian volume ≥ 10 mL [8, 10]. Pulsatile GnRH therapy has been tried in women with PCOS but the results remain mixed [11–13]. Neither the meta-analysis from the Cochrane Database [14], nor the Thessaloniki consensus [15], recommends GnRH pulsatile therapy in PCOS.

Besides PCOS, PCOM may also be observed as an isolated feature in about 30 % of normal women and in patients with FHA [16]. The association between FHA and PCOM has been poorly described in the literature and the studies are heterogeneous (variable diagnostic criteria for FHA and PCOM, confusion between hypogonadotropic hypogonadism and FHA, inconsistent LH level, small series or case reports) and as a result, the conclusions are contradictory [17–25]. More recently Dubourdieu et al. [26] have demonstrated the superiority of pulsatile GnRH therapy over gonadotropin, for ovulation induction in women with ‘FHA-PCOM’ (on-going pregnancy rates: 46.7 % versus 0 %; *p* = 0.02). However, this study has been restricted to only one treatment cycle, with interestingly similar ovarian responses in both groups.

It is therefore difficult to determine whether the presence of PCOM modifies or not the results of pulsatile GnRH therapy, when used for ovulation induction in women with FHA. Some issues remain unanswered such as: is there a higher risk of excessive ovarian response, of multi follicular responses and finally of multiple pregnancies? Or on the contrary, does the presence of PCOM make the stimulation more difficult, with lower ovulation and on-going pregnancy rates?

The aim of this study was to compare the use of pulsatile GnRH therapy for ovulation induction in patients with FHA and in patients with “FHA-PCOM”, and to obviate any possible impact of the presence of PCOM.

## Methods

This is a retrospective study in which data were collected from August 2002 to June 2015 in the Department of Endocrine Gynaecology and Reproductive Medicine, University Hospital of Lille, France. All women included were adults, wishing a pregnancy and presenting FHA only or “FHA-PCOM”. The patients were stimulated for ovulation induction with pulsatile GnRH therapy. This study was approved by the International Review Board of the University of Lille and all patients had given consent for the use of their clinical record.

### Population

Patients’ data, including their medical background, history of body weight, eating habits and the type and frequency of sports activity, were collected. Age, size, weight (with recent evolution) and body mass index were reported, as well as signs of hypometabolism (fatigue, chilliness, coldness of the body’s extremities, bradycardia, lanugo). All patients benefited from a full psycho-nutritional support, in order to identify any potential eating disorder. Patients with important eating disorders or metabolic complications were excluded from the study as their condition contraindicated pregnancy.

FHA was defined as secondary amenorrhea over 6 months, failure to bleed after progesterone withdrawal test, in a context of low body weight (BMI < 18) and/or history of important weight loss and/or intense physical activity, leading to a negative energy balance.. As FHA is a diagnosis of exclusion, every patient had a normal pituitary magnetic resonance imaging, normal prolactin and TSH levels and a non-elevated basal FSH level.

PCOM was defined by antral follicle excess on ultrasound and/or high serum AMH level. The threshold was ≥ 12 FNPO until 2008, then ≥ 19 FNPO with new ultrasound equipment (see below) [27]. Serum AMH level ≥ 35 pmol/L was considered as a surrogate to the excessive FNPO [27].

Ovulation induction was then carried out provided that the patient had a strictly normal hysterosalpingogram and the partner, a normal semen analysis.

Exclusion criteria were tubal obstruction, endometriosis, any other aetiology of central hypogonadism or sperm abnormalities.

### Assay and ultrasound procedures

For each patient, ultrasound and serum assays were performed a week after a progesterone challenge test (dydrogesterone 10 mg/day for 10 days). Patients remained in amenorrhea. Biological assessment included serums of FSH, E2, LH, prolactin, TSH, FT4, FT3, testosterone, dehydroepiandrosterone sulfate (DHEAS), Delta-4-androstenedione and 17-hydroxyprogesterone, as described previously [27–29]. Serum AMH levels were measured by ELISA technique with a second generation immunoassay kit, using monoclonal antibodies directed against the human AMH, called AMH-MIS (Beckmann Coulter, Immunotech Marseille, France), as previously described [27].

The ultrasound device used between 2002 and 2008 was a Logic 400 General Electric Milwaukee, replaced in 2008 by a General Electric Voluson E8 (probe frequencies were 5–7 MHz and 5–13 MHz, respectively). All follicles between 2 to 9 mm were included in the assessment of AFC.

### Treatment protocols

The GnRH pump (Lutrepulse®, Ferring, SAS, Gentilly, France) was placed intravenously (IV) or subcutaneously (SC) by the nurse of the department and was programmed to deliver 1 pulse of GnRH every 90 min (gonadoréline, Lutrelef® 3.2 mg, Ferring, SAS, Gentilly, France). The starting dose was the standard 15 μg SC and 5 μg IV, then adapted to the ovarian response.

The ovarian response was systematically controlled by serum E2 and LH assays and pelvic ultrasound. The first monitoring was made on the 8^th^ day of the stimulation, looking for multifollicular response and, if needed, treatment was adapted: decreased in case of multifollicular response or increased if no response. Patients were monitored under the same conditions until the emergence of at least one dominant follicle. The response was considered as excessive if there were more than 2 dominant follicles on ultrasound and/or E2 serum level ≥1835 pmol/L [30], leading to the cancellation of the cycle. Monitoring was ended once 1 or 2 dominant follicle(s) exceeded 13 mm in diameter. The pump remained in place until a spontaneous ovulation, as indicated by a serum progesterone assay (≥5 ng/mL) seven days after the presumed date of ovulation.

The luteal phase support was provided by one injection of hCG 1500 IU every 3 days, starting on the day of the withdrawal of the pump. A blood hCG pregnancy test was carried out systematically 14 days after ovulation. An ultrasound was performed around 6 or 7 weeks of amenorrhea in order to control the localisation of the pregnancy and its evolution, and to identify multiple pregnancies.

### Statistics

Quantitative variables were expressed as mean with standard deviation, or as median with 5^th^ and 95^th^ percentiles, according to the Gaussian or non-Gaussian distribution of the variables, respectively. To compare the two groups, the student T-test or the non-parametric Mann Whitney test was used, respectively. Qualitative variables were expressed in percentages and compared by a Chi^2^ or a Fisher test, according to the size of the population. Results were considered significant when *p* was < 0.05.

## Results

Sixty-seven patients were included in the study: 27 in the group “FHA” and 40 in the group “FHA-PCOM” (85 and 104 initiated cycles, respectively).

Both groups were similar except for the ultrasound aspect of PCOM and for serum AMH levels, which were significantly higher in “FHA-PCOM” patients (Table 1).

**Table 1:** Clinical, hormonal and ultrasound data

	FHA PCOM (*n* = 40)	FHA (*n* = 27)	*p*
Age (years)	28.5 [25.0–35.0]	28 [25.0–32.7]	NS
BMI (kg/m^2^)	18.5 [16.5–21.0]	18.1 [16.1–20.0]	NS
Clinical hyperandrogenism (%)	7.5 % (*n* = 3)	14.8 % (*n* = 4)	NS
LH (IU/L)	1.7 [0.5–3.9]	1.1 [0.5–3.6]	NS
FSH (IU/L)	4.9 [2.4–8.7]	5 [3.7–8.2]	NS
E2 (pmol/L)	80.7 [44.0–168.8]	73.4 [43.7–114.1]	NS
AMH (pmol/L)	51.0 [19.9–95.7]	18.4 [8.5–28.5]	<0,001
Follicle Number Per Ovary:			
- before 2008	13.0 [7.5–19.3]	7.5 [3.0–10.0]	<0,001
- after 2008	17.5 [9.8–41.0]	9.0 [3.0–14.3]	<0,001
Ovarian area (cm^2^)	4.1 [2.3–5.8]	3.1 [1.5–4.8]	<0,001
TESTOSTERONE (nmol/L)	0.7 [0.3–2.8]	0.7 [0.3–1.7]	NS
DELTA 4 (nmol/L)	4.5 [2.1–9.4]	4.2 [2.4–7.7]	NS
DHEAS (μmol/L)	3.4 [1.4–6.3]	3.4 [1.8–5.7]	NS

Values are medians with [5^th^–95^th^] percentiles

There was no significant difference concerning the ovulation rate (80.8 vs 77.7 %, NS), the cancelled cycle an excessive ovarian response rates (12.5 vs 10.6 %, NS) (Table 2). The dose of GnRH was not different between the groups, either for the first cycle or the mean of all cycles (Tables 2 and 4).

**Table 2:** Results per initiated cycles

	FHA PCOM (*n* = 104)	FHA (*n* = 85)	*p*
Ovulatory cycle rate	80.8 % (*n* = 84)	77.7 % (*n* = 66)	NS
Cancelled cycles rate	19.2 % (*n* = 20)	22.4 % (*n* = 19)	NS
- for excessive response	12.5 % (*n* = 13)	10.6 % (*n* = 9)	NS
- for poor response	1.9 % (*n* = 2)	0 % (*n* = 0)	NS
- for ovarian cyst	0 % (*n* = 0)	5.9 % (*n* = 5)	NA
- other	4.8 % (*n* = 5)	5.9 % (*n* = 5)	NS
Positive pregnancy test rate	32.7 % (*n* = 34)	24.7 % (*n* = 21)	NS
Clinical pregnancy rate	28.8 % (*n* = 31)	23.5 % (*n* = 20)	NS
Clinical ongoing pregnancy rate	26.9 % (*n* = 28)	20.0 % (*n* = 17)	NS
Miscarriage rate	2.9 % (*n* = 3)	3.5 % (*n* = 3)	NS
Multiple pregnancy rate	1.0 % (*n* = 1)	2.4 % (*n* = 2)	NS
Biochemical pregnancy rate	2.9 % (*n* = 3)	1.2 % (*n* = 1)	NS
Mean (+/− S.D) starting doses of GnRH chosen for the first cycle (μg):			
- intravenous	4.9 (+/− 0.3)	5.3 (+/− 1.3)	NS
- subcutaneous	12.0 (+/− 4.6)	15.0 (+/− 4.5)	NS
Mean (+/− S.D) starting doses of GnRH (all cycles) (μg):			
- intravenous	5.7 (+/− 2.1)	5.5 (+/− 2.1)	NS
- subcutaneous	11.8 (+/− 4.9)	13.1 (+/− 6.3)	NS

**Fig. 1:**
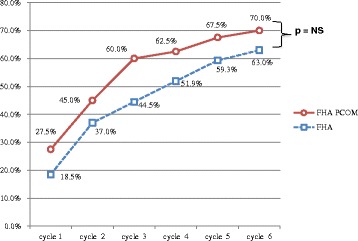
Cumulative on-going pregnancy rates per initiated cycle for FHA and FHA-PCOM patients

There was no significant difference concerning pregnancy rates, whether it was per initiated cycle (26.9 vs 20.0  %, NS; Table 2), or per ovulatory cycles (33.3 vs 28.5 %, NS; Table 3) or per patient (70.0 vs 63.0 %, NS; Fig. 1).

**Table 3:** Results per ovulatory cycles

	FHA PCOM (*n* = 84)	FHA (*n* = 66)	*p*
Positive pregnancy test rate	40.5 % (*n* = 34)	31.8 % (*n* = 21)	NS
Clinical pregnancy rate	36.9 % (*n* = 31)	30.3 % (*n* = 20)	NS
Clinical ongoing pregnancy rate	33.3 % (*n* = 28)	25.8 % (*n* = 17)	NS
Miscarriage rate	3.6 % (*n* = 3)	4.6 % (*n* = 3)	NS
Multiple pregnancy rate	1.2 % (*n* = 1)	3.0 % (*n* = 2)	NS
Biochemical pregnancy rate	3.6 % (*n* = 3)	1.5 % (*n* = 1)	NS

There was no significant difference between FHA and “FHA-PCOM” for biochemical pregnancy, miscarriage and multiple pregnancy rates.

There was no difference between the 2 groups when ovulatory cycles were compared (Table 4): same doses of GnRH, same stimulation duration, equivalent monofollicular response (78.6 vs 71.2 %, NS), and no more bifollicular reponse (20.2 vs 24.2 %, NS).

**Table 4:** Ovarian response in ovulatory cycles

	FHA PCOM (*n* = 84)	FHA (n = 66)	*p*
Stimulation duration (median, [5^th^–95^th^] percentiles)	18.9 [12.0–29.0]	18.1 [11.9–28.4]	NS
Mean (+/- S.D) total doses of GnRH (μg):			
- intravenous	117.8 (+/–51.7)	99.7 (+/–37.1)	NS
- subcutaneous	230.8 (+/–118.9)	286.3 (+/–175.3)	NS
Monofollicular response	78.6 % (*n* = 66)	71.2 % (*n* = 47)	NS
Bifollicular response	20.2 % (*n* = 17)	24.2 % (*n* = 16)	NS
Multifollicular response (≥2)	1.2 % (*n* = 1)	0 % (*n* = 0)	NS
Peri-ovulatory endometrium thickness (mm)	7.5 [4.0–11.0]	8.0 [3.5–11.4]	NS

## Discussion

This study shows that the groups were not different from each other, except for PCOM features and serum AMH. The gonadotropin insufficiency was therefore similar in our patients with FHA only and with “FHA-PCOM”. Ovulation induction with pulsatile GnRH therapy seemed to take place exactly the same way in both groups. Indeed, the doses of GnRH and the stimulation duration were equivalent. Ovarian response also appeared to be identical in both groups as there were the same monofollicular and bifollicular response rates. There was no more excessive ovarian response in patients with “FHA-PCOM”. And there was no difference between the two groups concerning on-going pregnancy rates, as well as miscarriage, multiple pregnancy and biochemical pregnancy rates.

The study observed about 20 % of bi-follicular response with pulsatile GnRH therapy in both groups. However and in agreement with the literature [4], the multiple pregnancy rate reported was low. The risk of multiple pregnancy is therefore not null, which imposes a fair information to couples, and a careful monitoring based on ultrasound and biology, rather than a simple clinical monitoring, as recommended in some previous studies [31].

This study demonstrates that the presence of PCOM in patients with FHA does not influence the management of the pulsatile GnRH therapy, nor the ovarian response to the treatment and the pregnancy rates. The pump appears therefore to be as efficient in “FHA-PCOM’” patient as it is in FHA patients [4]. This disagrees with previous studies [20, 22, 23] or case reports [17, 18], which showed the revelation of true PCOS in patients with “FHA-PCOM”, when treated with pulsatile GnRH therapy.

This study raises the question whether PCOM is actually indicative of a pre-existing but latent PCOS, “switched off” because of the gonadotropin insufficiency, or simply an ultrasound manifestation. The existence of PCOM is quite frequent in general population, including normo-ovulatory women with no hyperandrogenism [32–35]. In FHA, Robin et al. [16] identified (by cluster analysis) 3 groups of patients, depending on serum AMH level (as a surrogate for PCOM). The first two groups (normal serum AMH level (52 %) and moderate increase (about two-fold) of serum AMH level (38 %)) were in line with findings in female controls. The third group (10 %) with significantly higher serum AMH levels (about four-fold) was not found in controls. This suggests that this third group could match pre-existing PCOS “masked” by the gonadotropin deficiency of the FHA and would correspond to the cases reported by others [17, 20, 22, 23]. Our study is retrospective and cannot attest the revelation of true PCOS in some of our “FHA-PCOM” patients. To do so, it would be interesting to run a prospective trial and to systematically look for revelation of PCOS during the stimulation (elevation of serum androgens levels, increased number of follicles on ultrasound and/or serum AMH level…) [36].

The limitations of our study are mainly linked to its retrospective design. Although it might be the largest sample of patients with “FHA-PCOM” (40 patients, 84 initiated cycles), it remains a retrospective study with insufficient methodology to recommend pulsatile GnRH therapy as first-line treatment for ovulation induction. Also, the high prevalence of “FHA-PCOM” patients in this series was presumably due to a referral bias. Also, there are some controversies about the definition of PCOM, which is commonly defined by an excessive FNPO ≥ 12. However, this cut-off is highly dependent on ultrasound equipment and operator skill. Therefore, with the latest ultrasound generation, Dewailly et al. [27] have proposed a new threshold of 19 FNPO. Similarly, a panel of international experts has recently suggested a threshold of 25 follicles, when the ultrasound probe provides a maximum frequency greater that 8 MHz [37]. Serum AMH concentration is strongly correlated with the FNPO since it is mostly secreted from the small antral follicles from 2 to 9 mm, counted on ultrasound [37–40]. Dewailly et al. [27] have described a correlation between FNPO and serum AMH and defined serum AMH level ≥ 35 pmol/L as a surrogate for PCOM. However, this threshold was established with a specific centre and whether it can be extrapolated to other centres (using different control populations and AMH assays) still has to be verified. Last, the cost of one ovulatory cycle remains more expensive with the pump than with gonadotropins. However, in another study (under submission), we have compared the use of these two treatments for ovulation induction in “FHA-PCOM” patients and we showed that it was faster to induce a pregnancy with pulsatile GnRH therapy. Indeed, the on-going pregnancy rate after one initiated cycle of GnRH therapy was 28.9 %, while 3 subsequent cycles of gonadotropins had to be initiated to reach a close rate of 23.6 %. So, the total final cost for a pregnancy was relatively close between pulsatile GnRH therapy (1643€) and hMG (1421€), but much more expensive with recombinant gonadotropins (4334€). We did not take into account the costs of monitoring and blood samples (heavier with gonadotropins) and of eventual hospitalization for OHSS (more frequent with gonadotropins). As to the eventual discomfort of the pump, in our experience, most of the patients did not consider that carrying the device was worse than the daily injections of gonadotropins, where the stimulation duration was significantly longer.

## Conclusion

In conclusion, this study demonstrates that for ovulation induction, pulsatile GnRH therapy yields as good results in women having both FHA and PCOM as in women with FHA only. Therefore the presence of PCOM should not alter the management of FHA patients wishing a pregnancy. However, these findings need to be confirmed by prospective studies in order to make pulsatile GnRH therapy the first-line treatment for “FHA-PCOM” patients, just as it is for patients with FHA only.

### Ethics, consent and permissions

This study was approved by the International Review Board of the University of Lille and all patients had given consent for the use of their clinical record.
